# Exploring Gastric Perforation as an Uncommon yet Critical Complication of Gastrointestinal Stromal Tumors

**DOI:** 10.7759/cureus.58459

**Published:** 2024-04-17

**Authors:** Cielo S Silva-Ramos, Natalia M Barron-Cervantes, Alejandro Martinez-Esteban, Alejandro Alfaro-Goldaracena, Victor J Visag-Castillo

**Affiliations:** 1 General and Gastrointestinal Surgery Service, Fundación Clínica Medica Sur, Mexico City, MEX; 2 General and Oncological Surgery, Fundación Clínica Medica Sur, Mexico City, MEX

**Keywords:** roux-en-y gastric bypass, gastric perforation, gastrointestinal stromal tumor, gist, gastric tumor

## Abstract

Hollow viscus perforation poses a significant diagnostic and therapeutic dilemma for the majority of clinicians. It is vitally important that in cases of gastrointestinal perforation, the tissue that was perforated is always evaluated, since a malignant tumor can cause this complication as a presentation form. Here, we present the case of a patient whose first manifestation of a malignant gastric tumor was its perforation and the presence of septic shock secondary to this. This case exemplifies the importance of innovative thinking in facilitating a comprehensive diagnostic and therapeutic strategy, leading to the timely identification and management of a malignant tumor by the oncology team; such interventions not only enhance patient outcomes but also mitigate morbidity and mortality rates.

## Introduction

Gastrointestinal stromal tumors (GISTs) are malignant mesenchymal tumors of the gastrointestinal tract that are characterized by their formation from a common precursor of the interstitial nervous cell of Cajal and the smooth muscle cells. Despite representing the most common mesenchymal tumor of the digestive tract, GISTs represent less than 3% of all gastrointestinal neoplasms, and they will be characterized by the presence of a tyrosine kinase growth factor receptor, known as CD117 [1]. The term GIST was first used by Dr. Mazur and Dr. Clark to encompass a group of non-epithelial malignant tumors in the gastrointestinal tract that lacked the immunohistochemical characteristics associated with smooth muscle cells and Schwan cells; also, they present mutations in the KIT, specifically, c-KIT proto-oncogene. The importance of this mutation lies in the fact that the intracytoplasmic portion of KIT functions as a tyrosine kinase receptor, which is why these tumors are resistant to most chemotherapies. However, management with imatinib, a selective inhibitor of these receptors, is the treatment of choice, and since it began to be used as the first-line treatment, there has been a great improvement in the morbidity and mortality associated with GISTs [2]. 

The majority of these tumors are small and are likely to be asymptomatic. They tend to grow intramurally, but it is uncommon for them to cause dysphagia or bowel obstruction until they grow very large. The most common clinical presentation is with non-specific gastrointestinal symptoms, such as abdominal pain, nausea, and vomiting. On rare occasions, larger tumors have been associated with ulceration or even perforation. Diagnosis is made entirely by histopathology, including immunochemistry. Macroscopically, they are typically rounded tumors with frequent hemorrhage, which is why it explains that when seen through imaging studies, they present as rounded soft tissue masses presenting exophytic growth [3]. We present the case of a 73-year-old male patient who presented to the emergency room (ER) with abdominal pain intensity 10/10 that radiates to the thoracic region associated with abdominal distention and hemodynamic instability in a first-level private surgical center in Mexico City. This case is presented in order to further expand the knowledge about GISTs, as well as highlight the importance of a multidisciplinary approach in patients who present hemodynamically compromised and with data suggestive of abdominal perforation.

## Case presentation

A 73-year-old Latin American man presented to the ER with epigastric pain colic type, intensity 10/10 that radiates to the thoracic and anterior region of the neck, and hypogastrium, associated with significant abdominal distention. As relevant past medical history, the patient was diagnosed with systemic arterial hypertension in 2004, currently in pharmacological management with amlodipine and candesartan per oral (PO). He was also diagnosed with type 2 diabetes in the same year, currently in treatment with empagliflozin/linagliptin and glimepiride/metformin PO with no acute or chronic complications ever presented. Upon physical examination, he was tachycardic up to 131 rpm, tachypneic up to 26 rpm, and hypotensive with an arterial pressure of 99/69 mmHg. He presented abdominal pain located in the epigastric region to superficial and deep palpation, significant abdominal distention, and desaturation up to 80% at breathing room air. Also present was localized abdominal tenderness. During his stay in the ER, analgesic management was started with morphine 5 mg intravenously (IV), and a reservoir mask at 10 liters per minute was placed, with improvement in symptoms. General laboratory exams were solicited, in which leukocytosis at the expense of neutrophilia, hyperglycemia, elevation of creatinine and nitrogen, elevation of inflammatory markers, slightly elevated bilirubin, normal uncompensated GAP anion metabolic acidosis without meeting criteria for diabetic ketoacidosis, and hyperlactatemia were reported (Table 1).

**Table 1:** General laboratory exams performed at the ER. Laboratory tests included CBC, BC, clotting times, hepatic profile, and an arterial gasometry. PT: prothrombin time; INR: international normalized ratio; PTT: partial thromboplastin time; BUN: blood urea nitrogen; GPT: glutamic pyruvic transaminase; GOT: glutamic-oxaloacetic transaminase; GGT: gamma-glutamyl transferase; ALP: alkaline phosphatase; CRP: C-reactive protein; LDH: lactate dehydrogenase; CBC: complete blood count; BC: blood chemistry; ER: emergency room

Parameter	Value	Reference values
Hemoglobin	16.0 g/dL	12-18 g/dL
Medium corpuscular volume	82.2 fl	80-100 fl
Mean corpuscular hemoglobin	27.2 pg	23-31 pg
Hematocrit	49.1%	36-48%
Platelets	430x10^3^/uL	150-450x10^3^/uL
Leukocyte count	12.8x10^3^/uL	4.5-11x10^3^/uL
Absolute neutrophils	11.9x10^3^/uL	2.5-7x10^3^/uL
Absolute lymphocytes	0.6x10^3^/uL	1-4.8x10^3^/uL
PT	11.8 seconds	11-13.5 seconds
INR	1.1	1-1.1
PTT	29.8 seconds	25-35 seconds
Serum glucose	430.9 mg/dL	70-100 mg/dL
BUN	44.8 mg/dL	7-20 mg/dL
Serum creatinine	2.15 mg/dL	0.6-1.1 mg/dL
Serum sodium	132.7 mEq/L	135-145 mEq/L
Serum potassium	4.9 mEq/L	3.6-5.2 mEq/L
Serum chlorum	106 mEq/L	96-106 mEq/L
Serum calcium	8.7 mg/dL	8.5-10.5 mg/dL
Serum phosphorus	4.7 mg/dL	2.8-4.5 mg/dL
Serum magnesium	1.6 mg/dL	1.7-2.2 mg/dL
Albumin	3.2 g/dL	3.4-5.4 g/dL
Total bilirubin	1.75 mg/dL	1-1.2 mg/dL
Indirect bilirubin	1.23 mg/dL	0.2-1.2 mg/dL
Direct bilirubin	0.52 mg/dL	0-0.35 mg/dL
GPT	34 U/L	4-36 U/L
GOT	40 U/L	5-40 U/L
GGT	9.7 U/L	0-30 U/L
ALP	84 U/L	44-147 U/L
CRP	265.9 mg/dL	<0.3 mg/dL
LDH	187 U/L	140-280 U/L
pH	7.35	7.35-7.45
pCO2	27.9 mmHg	35-45 mmHg
pO2	85.2 mmHg	75-100 mmHg
Lactate	3.2 mmol/L	<1.0 mmol/L
HCO3	15.6 mEq/L	22-28 mEq/L
Anion gap	15.9 mmol/L	4-12 mmol/L

**Figure 1:**
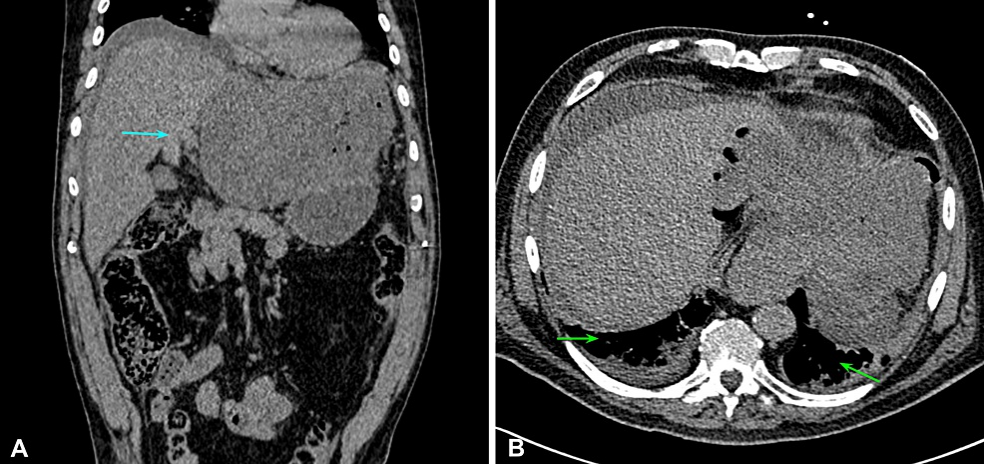
Non-contrasted abdominal CT scan. Esophagogastric junction with prominence of gastric folds through the hiatus: peritoneal free air with perihepatic and perisplenic free fluid is observed (green arrows). A heterogeneous image with air that seems to depend on the lesser curvature of the stomach that loses interface and measures approximately 10x15x12 cm (blue arrow) associated with multiple lymph nodes and peritoneal fatty striation: part of the stomach, body, and antrum are seen displaced downwards. Image A: coronal plane. Image B: axial plane.

Because of the clinical presentation, a non-contrasted abdominal CT scan was requested, which reported data compatible with an ulcerated tumor dependent on the lesser curvature of the stomach associated with air and free liquid (Figure 1).

**Figure 2:**
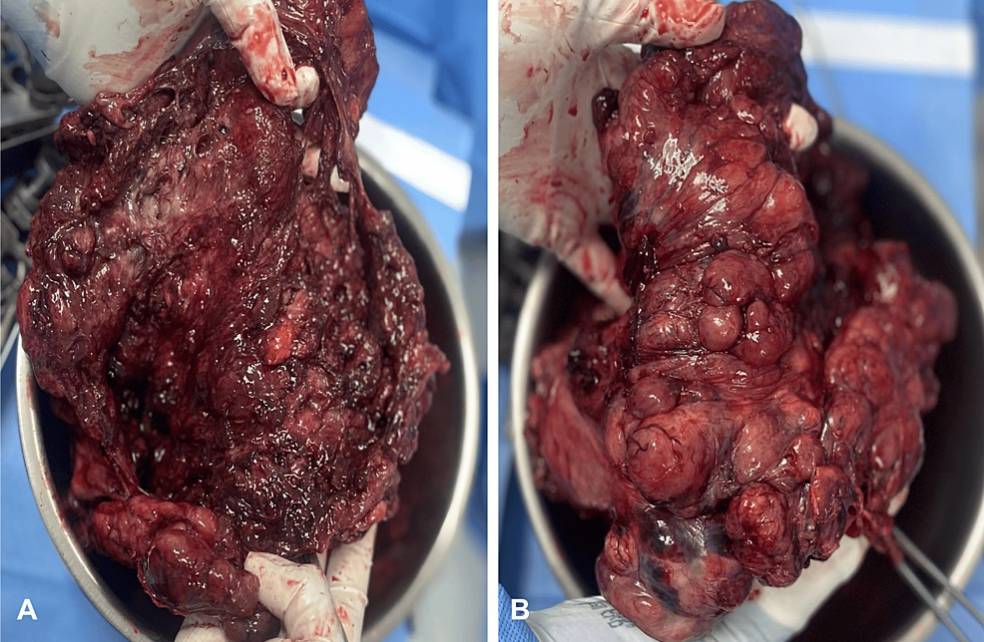
Transoperative images. Image A: exophytic mass at the expense of the gastric fundus. Image B: gastroesophageal junction showing part of the exophytic mass.

The diagnosis of grade I septic shock due to an abdominal focus secondary to gastric perforation is integrated. The patient was hospitalized at the intensive care unit (ICU) and started an antibiotic regimen based on meropenem IV, vancomycin IV, and anidulafungin IV. The patient underwent an exploratory laparotomy. During this surgery, a midline incision was performed. Upon entering the abdominal cavity, the cavity and abscess were washed and drained, and these collections were sent for cultures. During this exploration, an exophytic mass was identified at the expense of the anterior surface of the superior part of the stomach, dependent on the gastric fundus. The tumor directly invaded the liver segment II and the diaphragm with an extent of invasion approximately 1 cm each. Because of this, a total gastrectomy, distal esophagectomy, and resection of the affected diaphragm were executed (Figure 2). As the diaphragm resection was only about 1 cm, a primary closure of the defect was performed, and no mesh was required. The intraoperative histopathological study was found with disease-free edges.

Subsequently, restitution of esophageal transit was performed using a mechanical esophagojejunal anastomosis and Roux-en-Y reconstruction. A non-anatomical hepatectomy was performed where a portion of liver segment II of approximately 1x1 cm was resected to be sent for histopathological study. Finally, a left chest tube and a lower left quadrant abdominal Blake drainage were placed. The final histopathology report reported a >10 cm perforated exophytic tumor composed of spindle or epithelioid cells that in immunochemistry was positive for CD117 and CD34 with a high mitotic index (≥5/50 per high-power field), presenting the final diagnosis of an epithelioid/mixed phenotype of GIST with a high risk according to the National Institutes of Health (NIH)-Fletcher criteria for GIST risk assessment [4]. 

After surgery, the patient returned to the ICU for close postoperative monitoring, where he stayed for three days. Final abscess cultures reported *Klebsiella pneumoniae* and *Candida albicans*, sensitive to the treatment previously mentioned, which is why during his hospitalization, the patient completed this antibiotic scheme with a favorable postoperative evolution. The patient remained hemodynamically stable in intensive care and was transferred to intermediate therapy, starting oral administration of pharmacological treatments and diet with adequate tolerance. When he showed clinical improvement, he was transferred to a regular hospitalization floor and was discharged without incident after a week. According to the surgical, imaging, and histopathological findings, the patient status was determined to be T4 N0 M0, as the final histopathological report from both the liver and the diaphragmatic lesions were negative for GIST and no lymphatic involvement was seen during surgery. This resulted in the clinical stage being IIIB according to GIST staging classification by the American Joint Committee on Cancer (AJCC) TNM system [5]. Due to the high risk of recurrence, adjuvant management with imatinib, a tyrosine kinase inhibitor, was indicated for at least three years with close follow-up by the oncology and oncologic surgery team. One year post-surgery, the patient is in good general condition without tumor recurrence and presents asymptomatic.

## Discussion

GISTs are the most common mesenchymal malignant tumor on the gastrointestinal tract, most commonly seen in 60-year-old people with an equal gender distribution. On average, 18% were diagnosed as incidentalomas. The most frequent location is the stomach with 55-70%, followed by the small intestines with 30%, colorectal with 6-7%, and esophagus being less than 1%. The highest incidence rates in the world are reported in China, Taiwan, and Norway [6]. As previously mentioned, they are usually small asymptomatic tumors. However, they are commonly associated with exophytic growth and erosion of the gastrointestinal tract lumen which results in ulceration, hemorrhage, and perforation, as seen in the case presented here. Patients with GISTs may also present non-specific abdominal symptoms as abdominal pain, distension, early satiety, and anemic syndrome related to hemorrhage. This clinical presentation is very important because as it presents completely asymptomatic or with non-specific symptoms until it grows and starts to present complications, between 10% and 25% of all cases present with metastatic disease at the diagnosis. Intra-abdominal metastases in the liver, peritoneum, and mesenteric and other serosal surfaces are the most common, while extra-abdominal or lymphatic metastases are rarely presented which is why if not suspected macroscopically, lymphadenectomy is not indicated [2]. Referred pain can be demonstrated in this case, where the patient presented with epigastric pain that radiated to the thoracic and anterior region of the neck; this phenomenon arises due to the innervation patterns of dermatomes by distinct peripheral nerves, and it is commonly classified as either radicular pain or neuropathic pain. This atypical presentation presents a diagnostic challenge as the clinical presentation does not represent the anatomical region directly affected, which can delay timely diagnosis if referred pain is not suspected [7].

Even though final diagnosis cannot be achieved through non-invasive imaging studies, the use of abdominal CT scan has been proved to be useful as part of the preoperative approach. In general, these tumors present as rounded tissues compatible with soft tissue masses arising from the gastrointestinal growth with an endoluminal or exophytic growth, mostly being the second. The usual growth pattern presented was exoenteric with a large extraluminal component. Also, mucosal ulceration can be seen as necrotic communicating cavities between the gastrointestinal tract and the abdominal cavity, seen in almost 50% of all cases [8]. The final diagnosis can be reached through a histopathological study. Macroscopically, they present as small rounded tumors, but if they are larger, they may present as cystic tumors, which typically on sectioning show a pinkish-tan, fish flesh-like content and present with hemorrhage. Histologically, they engage a wide group of mesenchymal tumors that may present as spindle cell or epithelioid tumors, with sclerosing spindle cell being the most commonly seen morphology. However, palisaded-vacuolated morphology is the most common in gastric GISTs [9]. These tumors present with a low cellularity and a high content of collagenous matrix that may or may not present calcifications. In general, they present as small tumors with lower mitotic rates, being tumors <2 cm with mitotic rates ≤5/50 high-power fields (5 mm^2^ total area). These characteristics are associated with a favorable prognosis, but if these are not fulfilled, they have a significant tumor/related mortality [9]. 

To complement the histological study, immunohistochemistry should always be performed. In here, the cells will show positive results for CD117 and CD34 mainly; however, other markers such as Anoctamin 1 and KIT have been described. Despite KIT mutations being the most commonly seen, sometimes platelet-derived growth factor receptor alpha (PDGFRA) mutations are shown instead. These two genes are homologous as they evolved as a duplication from a common ancestral gene. The importance of identifying the specific mutation presented in each tumor is that tumors with PDGFRA mutations have been associated with lesser imatinib sensitivity [10]. Surgery is the gold standard management for all non-metastatic GISTs with no lymph node resection. When performing this surgery, the goal is to remove the tumor as a block, including its pseudocapsule, and to utilize intraoperative histopathological studies to prove adequate resection margins. The ultimate therapeutic goal in primary GISTs is to achieve negative resection margins, also known as R0, always seeking the greatest preservation of the organ from which the tumor originates, as well as avoiding rupturing the tumor [11]. Then, the use of an adjuvant therapy is needed, in this case being imatinib. This pharmacological treatment was initially developed to treat chronic myeloid leukemia (CML) by inhibiting the fusion of the BCR-ABL protein. Nowadays, imatinib is considered the first-line treatment in metastatic, recurrent, or residual GIST with an observed response of 65-70% and a median response of duration that exceeds two years. Adjuvant therapy is only indicated in patients at a high risk of recurrence with a treatment going from one to three years depending on the progression of the disease. Response monitoring can be carried out by the use of abdominal CT scan, MRI, and/or metabolic imaging with fluorodeoxyglucose-positron emission tomography (FDG-PET), depending on the patient and clinician preferences [12,13].

## Conclusions

GISTs represent a rare tumor that usually presents as perforation or intra-abdominal bleeding. It is vitally important to define the origin of all intestinal perforations since GISTs are a cause that, despite being rare, must always be ruled out to avoid the progression of the disease. This case demonstrates that a multidisciplinary approach that includes the oncological surgery team allows for early identification as well as timely treatment that reduces the morbidity and mortality of patients. Particularly in this case, it is important to emphasize the ingenuity of preoperative diagnosis and surgical intervention, as suspicion regarding the gastric origin of referred chest pain allowed for targeted abdominal exploration, leading to the identification of secondary hemorrhage due to gastric perforation. The surgical strategy pursued was conservative, aiming for minimal resection while ensuring negative margins. This patient serves to observe the correct approach that led to a correct and early identification. The surgery performed and described here allowed the surgeon to achieve an R0 resection, the goal in these cases, but also to identify the high risk of recurrence and thus be able to use adjuvant therapy with imatinib.
